# Complete Nucleotide Sequence of Porcine Circovirus 2 Isolated from Piglets in China

**DOI:** 10.1128/genomeA.00274-13

**Published:** 2013-07-18

**Authors:** Xiaoliang Hu, Zhige Tian, Bo Peng, Hui Chen, Juanjuan Qu

**Affiliations:** Northeast Agricultural University, Harbin, People's Republic of Chinaa; Northeast Forestry University, Harbin, People's Republic of Chinab; Jilin Jingxiang Animal’s Pharmaceutical Company, Jilin, People’s Republic of Chinac; Heilongjiang Hongqiao Hospital, Harbin, People's Republic of Chinad

## Abstract

A new strain of canine distemper virus, strain HX, has been isolated from piglets in China, and its complete genome has been sequenced and analyzed. Phylogenetic analysis suggests that HX belongs to the 2d genotype cluster and it is highly prevalent in China.

## GENOME ANNOUNCEMENT

Porcine circovirus 2 (PCV2), which belongs to the family *Circoviridae*, is a nonenveloped, single-stranded circular DNA virus with a genome of about 1,766, 1,767, or 1,768 bp, depending on the genotype. Its genome includes replicase (*rep*) and capsid (*cap*) genes for the two major open reading frames (ORFs) and a virus-induced apoptotic gene, ORF1, ORF2, and ORF3, respectively (1). PCV2 is suspected to be associated with a clinical condition observed in pigs shortly after weaning and fattening known as postweaning multisystemic wasting syndrome (PMWS), which was first identified in Canada during the early 1990s (2). Sequence analyses revealed that PCV2 isolates can be clustered into 5 genotypes, designated 2a, 2b, 2c, 2d, and 2e (3, 4).

In this study, PCV2 strain HX was detected and identified in the organs of piglets that were showing clinical signs of PMWS. Tissue samples were collected, minced, and pooled into a 20% suspension with Dulbecco’s modified Eagle medium (DMEM). The DNA was then extracted from the supernatant of each suspension using a commercial DNA extraction kit (Omega) according to the manufacturer’s instructions and analyzed with PCR by using a primer pair (forward, ATCCACGGAGGAAGGGGGGCCAGTT, and reverse, GTGGATTGTTCTGTAGCATTCTTCCA) that amplified a 1.7-kb fragment, which indicated that the infecting agent was PCV2. The PCR product was cloned into the pMD18-T vector (TaKaRa) and sequenced using an ABI3730xl Sanger-based genetic analyzer. Sequences were compiled using the SeqMan program in Lasergene. The genome of the HX strain is 1,767 nucleotides (nt) and consists of three ORFs. The levels of identity between the complete sequence of the HX strain and those of 9 other PCV2s ranged from 95.3% for PCV2 strain LG (GenBank accession no. HM038034) to 99.9% for PCV2 strain AH (GenBank accession no. HM038030). The levels of identity for the *rep* gene ranged from 96.3% for PCV2 strain SH (GenBank accession no. HM038027) to 99.5% for strain AH, and those for the *cap* gene ranged from 91.9% for PCV2 strain CL (GenBank accession no. HM038034) to 100% for strain LG. Phylogenetic analysis and multiple sequence alignment according to the complete nucleotide sequence revealed that HX belongs to the PCV2d cluster, which is a novel genotype in China.

The complete sequence of the HX strain permitted a detailed analysis of the genetic variation of PCV2s in China that is likely to be helpful to guide efficient diagnostic, preventive, and control strategies for canine distemper virus (CDV) in China. In addition, these data will be helpful for analyses of the evolutionary characteristics and molecular pathogenesis of PCV2.

### Nucleotide sequence accession number.

The genome sequence of the HX strain has been submitted to GenBank under the accession no. KC860786.
